# AgNPs@CeO_2_/Nafion Nanocomposite-Modified Electrode for the Sensitive Detection of Trace Lead (II) in Water Samples

**DOI:** 10.3390/s25092655

**Published:** 2025-04-23

**Authors:** Zhengying Guo, Peng Xu, Shiqing Zhou, Ruoxi Wu

**Affiliations:** 1 Hunan Engineering Research Center of Water Security Technology and Application, College of Civil Engineering, Hunan University, Changsha 410082, China; 2 Department of Water Engineering and Science, College of Civil Engineering, Hunan University, Changsha 410082, China

**Keywords:** lead (II), sensor, nano-ceria (CeO_2_), AgNPs, Nafion-modified electrode

## Abstract

Excessive levels of heavy metal pollutants in the environment pose significant threats to human health and ecosystem stability. Consequently, the accurate and rapid detection of heavy metal ions is critically important. A AgNPs@CeO_2_/Nafion composite was prepared by dispersing nano-ceria (CeO_2_) in a Nafion solution and incorporating silver nanoparticles (AgNPs). The morphology, microstructure, and electrochemical properties of the modified electrode materials were systematically characterized using scanning electron microscopy (SEM), energy-dispersive X-ray spectroscopy (EDX), X-ray diffraction (XRD), and cyclic voltammetry (CV). By leveraging the oxygen vacancies and high electron transfer efficiency of CeO_2_, the strong adsorption capacity of Nafion, and the superior conductivity of AgNPs, an AgNPs@CeO_2_/Nafion/GCE electrochemical sensor was developed. Under optimized conditions, trace Pb^2+^ in water was detected using square wave anodic stripping voltammetry (SWASV). The sensor demonstrated a linear response for Pb^2+^ within the concentration range of 1–100 μg·L^−1^, with a detection limit of 0.17 μg·L^−1^ (S/N = 3). When applied to real water samples, the method achieved recovery rates between 93.7% and 110.3%, validating its reliability and practical applicability.

## 1. Introduction

Lead–zinc mining and metal smelting are primary contributors to environmental lead pollution [1]. As a non-degradable heavy metal, the excessive accumulation of lead in ecosystems poses direct and indirect threats to environmental health. Lead is a potent neurotoxin; overexposure in humans can result in cognitive decline, learning disabilities, developmental delays, and irreversible damage to the nervous, skeletal, circulatory, enzymatic, endocrine, and immune systems [2]. In recent years, there has been a notable increase in reports of lead poisoning incidents and elevated blood lead levels globally, heightening concerns about lead contamination [3,4,5]. Therefore, it is imperative to develop rapid, accurate, and sensitive techniques for detecting lead in aquatic environments to enhance environmental monitoring and protect public health.

In the field of heavy metal detection, traditional analytical methods such as inductively coupled plasma mass spectrometry (ICP-MS) [6], atomic emission spectroscopy [7], atomic absorption spectroscopy [8], and X-ray fluorescence spectroscopy [9] are widely recognized for their efficacy. However, these techniques necessitate expensive and cumbersome equipment and specialized operators and incur significant per-sample costs. In contrast, electrochemical detection represents a cost-effective, time-efficient, user-friendly, and field-deployable method, offering a promising alternative for the analysis of heavy metal ions [10]. Currently, extensive research has been carried out on electrochemical detection technology for lead ions. Nevertheless, its practical application still encounters challenges, including sensitivity, inadequate anti-interference capability, insufficient stability, and difficulties in cost management [11]. Akbari et al. [12] synthesized a bidentate Schiff base ligand and conjugated it with gold nanoparticles to fabricate the AuNPs-L1/SPCE sensor for detecting Pb^2+^ in seawater. While this sensor exhibits high sensitivity (56.78 μA μM^−1^ cm^−2^) and environmental applicability, its detection limit is 0.298 μM. This makes it challenging to meet the requirements for trace-level detection. Additionally, cost considerations remain a significant factor limiting the practical implementation of this technology. Zhang et al. [13] prepared the AuNPs/PPy/Ti_3_C_2_T_x_ nanocomposite sensor by the layer-by-layer self-assembly method. Through the Au-S bond bioconjugation and the stabilizing effect of polypyrrole, the detection limit of Pb^2+^ was reduced to 1 × 10^−14^ M, and the linear range spanned five orders of magnitude (5 × 10^−14^ to 1 × 10^−8^ M). However, this technology relies on precious metal AuNPs and MXene materials, with high raw material costs and complex preparation processes, which seriously restricts the potential for large-scale production.

The modification of electrodes using nanomaterials has emerged as an effective strategy to enhance performance and functionality. Nano-metal oxides are widely utilized as electrode modifiers due to their superior adsorption capacity, larger specific surface area, and enhanced electron transfer kinetics [14]. CeO_2_, a cost-effective and versatile light rare-earth oxide, functions as an n-type semiconductor with a unique 4f electronic structure and abundant surface oxygen vacancies. The Ce^3+^/Ce^4+^ redox couple further facilitates electron mobility, endowing CeO_2_ with exceptional redox activity and improved electron transfer efficiency [15,16]. Nevertheless, the application of CeO_2_ in heavy metal ion sensors remains at an exploratory stage. Based on a comprehensive review of the literature, research reports on CeO_2_-based materials for heavy metal ion detection are currently scarce. For instance, Sun et al. [16] developed oxygen-rich vacancy ruthenium-loaded cerium dioxide nanocomposites that demonstrated highly sensitive electrochemical detection of Hg^2+^ with excellent anti-interference performance even in the presence of other heavy metal ions (Cd^2+^, Cu^2+^, Pb^2+^). Cui et al. [17] engineered a CeFe_2_O_5_ nanocomposite by incorporating iron ions into the cerium oxide lattice, which enhanced both adsorption and redox processes. This CeFe_2_O_5_/GCE sensor exhibited a detection limit of 0.94 nM for Cd^2+^. Rani et al. [18] fabricated nano-cerium oxide using orange peel extract via an environmentally friendly approach and detected Cd^2+^ through SWASV. The method achieved a linear detection range of 10 μM to 1000 μM, with a detection limit of 0.47 nM and good stability. Singh et al. [19] pioneered the synthesis of cerium oxide-catalyzed one-dimensional carbon nanofibers grown by chemical vapor deposition using the tip growth method for the simultaneous detection of Pb^2+^ and Cu^2+^. The resulting Ce-CNF electrode showed favorable linear relationships for Pb^2+^ and Cu^2+^, with detection limits of 0.6 ppb and 0.3 ppb, respectively.

Furthermore, the synergistic enhancement of sensing performance can be achieved by introducing noble metal nanoparticles (such as AgNPs) into CeO_2_-based materials to construct heterogeneous junction composite materials. AgNPs, with their high surface area, exceptional conductivity, and electrocatalytic activity [20], have been extensively utilized in supercapacitors [21] and electrochemical sensors [22]. It is worth noting that research on AgNPs@CeO_2_ composites has advanced significantly in areas such as the photocatalytic degradation of organic pollutants [23], optics [24], and antibiosis [25] in recent years. However, their application in the sensor field remains largely confined to gas sensing [26], immunosensors [27], and the detection of organic molecules [28], with no reported instances of their use for detecting heavy metal ions. This application gap presents an innovative opportunity for this study.

In this study, Nafion was innovatively incorporated into the AgNPs@CeO_2_ system. By employing a straightforward synthesis method, a low-cost yet high-performance ternary composite sensor of AgNPs@CeO_2_/Nafion/GCE was successfully fabricated. Nafion, a perfluorosulfonic acid copolymer, contains numerous hydrophilic ionized sulfonic acid groups, enabling efficient cation exchange and heavy metal ion accumulation on the electrode surface. This mechanism significantly improves sensor sensitivity and selectivity. Moreover, Nafion’s excellent mechanical stability and aqueous compatibility make it an ideal matrix for immobilizing modifiers [29,30]. Furthermore, Nafion, serving as a dispersion carrier, effectively suppresses the agglomeration of AgNPs. Experimental results demonstrate that, in comparison to the pure CeO_2_ substrate, the CeO_2_/Nafion composite substrate markedly enhances the uniformity of AgNPs dispersion. The synergistic interaction among CeO_2_, Nafion, and AgNPs has established a sensitive platform for lead detection. This study underscores the promising potential of rare-earth elements, exemplified by CeO_2_, in lead sensing and advances the broader field of heavy metal detection, with significant implications for environmental monitoring and water safety.

## 2. Materials and Methods

### 2.1. Instruments and Reagents

Anhydrous ethanol (C_2_H_5_OH), potassium chloride (KCl), potassium ferricyanide (K_3_[Fe(CN)_6_]), sodium acetate (CH_3_COONa, NaAc), acetic acid (CH_3_COOH, HAc), sodium hydroxide (NaOH), hydrochloric acid (HCl), sodium dihydrogen phosphate (NaH_2_PO_4_), sodium hydrogen phosphate (Na_2_HPO_4_), and silver nitrate (AgNO_3_) were purchased from Sinopharm Chemical Reagent Co., Ltd. (Shanghai, China). Cerium (III) nitrate hexahydrate (Ce(NO_3_)_3_·6H_2_O), Triton X-100, sodium dodecyl sulfate (SDS), cetyltrimethylammonium bromide (CTAB), ascorbic acid (AA), lysine (Lys), alanine (Ala), and Nafion perfluorinated resin solution (5 wt%) were obtained from Shanghai Macklin Biochemical Technology Co., Ltd. (Shanghai, China). The Pb^2+^ standard solution (100 μg·mL^−1^), zinc sulfate heptahydrate (ZnSO_4_·7H_2_O), calcium chloride dihydrate (CaCl_2_·2H_2_O), ferric chloride hexahydrate (FeCl_3_·6H_2_O), copper (II) sulfate pentahydrate (CuSO_4_·5H_2_O), potassium nitrate (KNO_3_), sodium chloride (NaCl), magnesium chloride hexahydrate (MgCl_2_·6H_2_O), cadmium chloride (CdCl_2_), and manganese sulfate monohydrate (MnSO_4_·H_2_O) were supplied by Shanghai Aladdin Biochemical Technology Co., Ltd. (Shanghai, China). An acetate buffer solution (HAc-NaAc, ABS, 0.1 M) with varying pH levels was prepared as the supporting electrolyte by mixing appropriate volumes of 0.1 M NaAc and HAc solutions. Phosphate buffer solution (PB, 0.1 M) was prepared by mixing different proportions of 0.1 M NaH_2_PO_4_ and Na_2_HPO_4_ stock solutions. The pH was measured using a calibrated pH meter and precisely adjusted to 4.5 through the dropwise addition of either 0.1 M NaOH or 0.1 M HCl as needed. A 0.1 M HCl solution was titrated with 0.1 M NaOH until the target pH of 4.5 was achieved. All chemicals used were of analytical grade and directly usable without further purification. All solutions were prepared using ultrapure water (18.2 MΩ·cm).

Electrochemical measurements were conducted using a CHI660E electrochemical workstation (CH Instruments, Shanghai, China) with a standard three-electrode system: a glassy carbon electrode (GCE, 3 mm diameter) as the working electrode, a platinum plate functioned as the counter electrode, and a Ag/AgCl electrode acted as the reference electrode. The morphology of the materials was characterized using a field-emission scanning electron microscope (SEM, ZEISS Sigma 300, Oberkochen, Germany). The crystal structure and elemental composition were analyzed by X-ray diffraction (XRD, Rigaku SmartLab SE, Tokyo, Japan). The concentration of lead ions in real water samples was cross-validated and quantitatively analyzed using ICP-MS (Agilent 7700x, Santa Clara, CA, USA).

### 2.2. Synthesis of CeO_2_ Nanoparticles

Nano-CeO_2_ particles were synthesized using a hydrothermal method. Briefly, Ce(NO_3_)_3_·6H_2_O (0.868 g) and NaOH (0.016 g) were separately dissolved in 5 mL and 35 mL of distilled water, respectively, under continuous stirring. The NaOH solution was subsequently added dropwise to the Ce(NO_3_)_3_ solution, and the resulting mixture was stirred at room temperature for 30 min. Subsequently, the solution was transferred into a Teflon-lined stainless-steel autoclave and maintained at 100 °C in a drying oven for 24 h. Upon cooling to room temperature, the product was centrifuged and washed multiple times with anhydrous ethanol and deionized water to remove unreacted precursors. Finally, the resulting white solid was dried in a vacuum oven at 60 °C for 12 h and then ground into a fine powder for further use.

### 2.3. Fabrication of AgNPs@CeO_2_/Nafion/GCE

#### 2.3.1. Electrode Pre-Treatment

The GCE was sequentially polished using 0.3 μm and 0.05 μm alumina polishing powder by drawing a figure “8” on chamois leather. The polished GCE was then subjected to electrochemical validation through CV in a KCl/K_3_[Fe(CN)_6_] solution until acceptable surface cleanliness was achieved. Subsequently, the electrode was ultrasonically cleaned for 30 s each in nitric acid solution (1:1 *v/v* HNO_3_/H_2_O), anhydrous ethanol, and ultrapure water, followed by drying under a nitrogen flow.

#### 2.3.2. Modification with CeO_2_/Nafion Composite

A 0.25 wt% Nafion solution was prepared by diluting the original 5 wt% Nafion stock solution with anhydrous ethanol. The synthesized CeO_2_ nanoparticles were then ultrasonically dispersed into this diluted Nafion solution to obtain a final concentration of 2 mg·mL^−1^ CeO_2_ in the suspension. Subsequently, a 5 μL aliquot of the CeO_2_/Nafion suspension was drop-casted onto the bare GCE surface and air-dried at room temperature.

#### 2.3.3. Electrodeposition of AgNPs

The CeO_2_/Nafion/GCE was immersed in a deposition solution containing 1 mmol/L AgNO_3_ and 100 mmol/L KNO_3_. AgNPs were electrodeposited via CV under the following parameters: potential window from −0.3 to 1.2 V, 16 scan cycles, and scan rate of 50 mV/s. After deposition, the modified electrode was rinsed thoroughly with ultrapure water and dried at room temperature.

### 2.4. Electrochemical Testing

The three-electrode system was constructed using a modified glassy carbon electrode as the working electrode, a platinum plate as the counter electrode, and an Ag/AgCl electrode as the reference electrode. The electrochemical performance of the modified electrode was evaluated via CV. The CV measurements were performed in a solution containing 5 mM K_3_[Fe(CN)_6_]/K_4_[Fe(CN)_6_] redox couple and 0.1 M KCl supporting electrolyte at a scan rate of 50 mV·s^−1^.

SWASV was employed for the detection of Pb^2+^ ions. A 0.1 mol·L^−1^ HAc-NaAc buffer solution (pH 4.5) served as the supporting electrolyte. A specific concentration of Pb^2+^ solution was introduced into the electrolyte system, followed by purging with high-purity nitrogen gas for 10 min to eliminate dissolved oxygen. The SWASV parameters were set as follows: deposition potential of −1.1 V, deposition time of 300 s, potential window ranging from −0.8 V to −0.2 V, frequency of 15 Hz, amplitude of 25 mV, and potential increment of 4 mV. Magnetic stirring was applied during the deposition phase, while stirring was halted during the stationary and dissolution stages. All experiments were conducted under ambient temperature conditions.

### 2.5. Water Sample Preparation

This study selected four representative water samples, namely tap water, river water, rainwater, and mineral water, to comprehensively evaluate the practical detection capability of AgNPs@CeO_2_/Nafion/GCE for Pb^2+^. Tap water was obtained from the laboratory water supply system, river water samples were collected from the Xiangjiang River (Changsha, China), rainwater was accumulated outdoors on campus, and mineral water was commercially available natural mineral water. All water samples were first filtered using 0.22 μm syringe filters to remove suspended particulates and microbial biomass, and subsequently subjected to acid digestion. The digested water samples were then employed in lieu of ultrapure water for the preparation of a pH 4.5, 0.1 M HAc-NaAc buffer solution. Standard solutions of Pb^2+^ with different concentration gradients were sequentially added to the pre-treated water samples. Subsequently, standard addition recovery experiments were conducted by SWASV to calculate the recovery rate (Recovery), thereby evaluating the actual detection capability of AgNPs@CeO_2_/Nafion/GCE. To ensure data reliability, ICP-MS was employed for quantitative analysis. The specific procedure is as follows: The instrument was calibrated for accuracy, and parameters were optimized using the tuning solution. A gradient standard solution of lead ions was prepared to construct the calibration curve. Signal drift and matrix interference were corrected by incorporating the rhodium (Rh) internal standard solution. The pre-treated samples were analyzed concurrently with the standard solutions, and the lead concentration was determined based on the calibration curve. All experiments were performed under identical environmental conditions, with six parallel measurements conducted at each concentration level.

## 3. Results

### 3.1. Characterization of Electrode Materials

**Figure 1:**
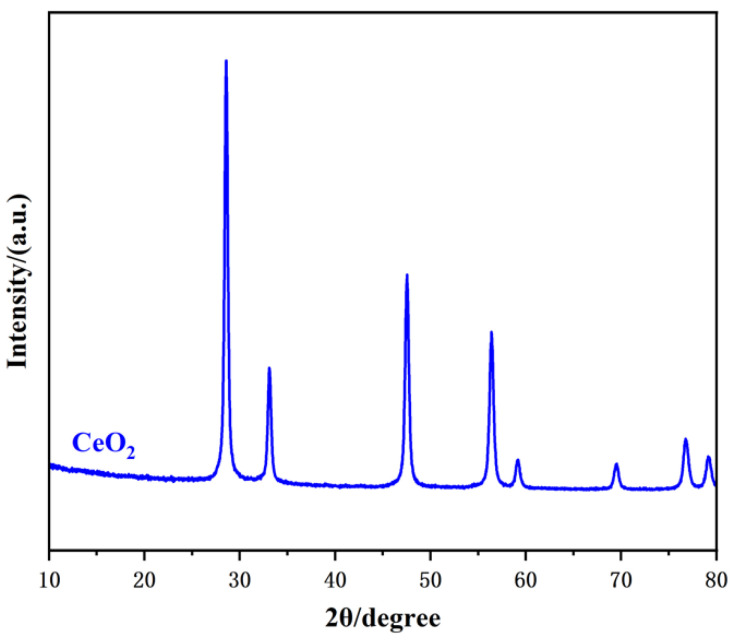
XRD pattern of the CeO_2_ sample.

The crystal structure of CeO_2_ was characterized using XRD. As illustrated in Figure 1, the XRD pattern of CeO_2_ displays distinct diffraction peaks at 2θ = 28.5°, 33.1°, 47.5°, 56.4°, 59.1°, 69.5°, 76.7°, and 79.3°, corresponding to the (111), (200), (220), (311), (222), (400), (331), and (420) crystal planes. These diffraction peaks are in agreement with the standard fluorite structure (JCPDS Card No. 34-0394) and are consistent with previous reports [15,31,32], thereby confirming the successful synthesis of polycrystalline CeO_2_.

**Figure 2:**
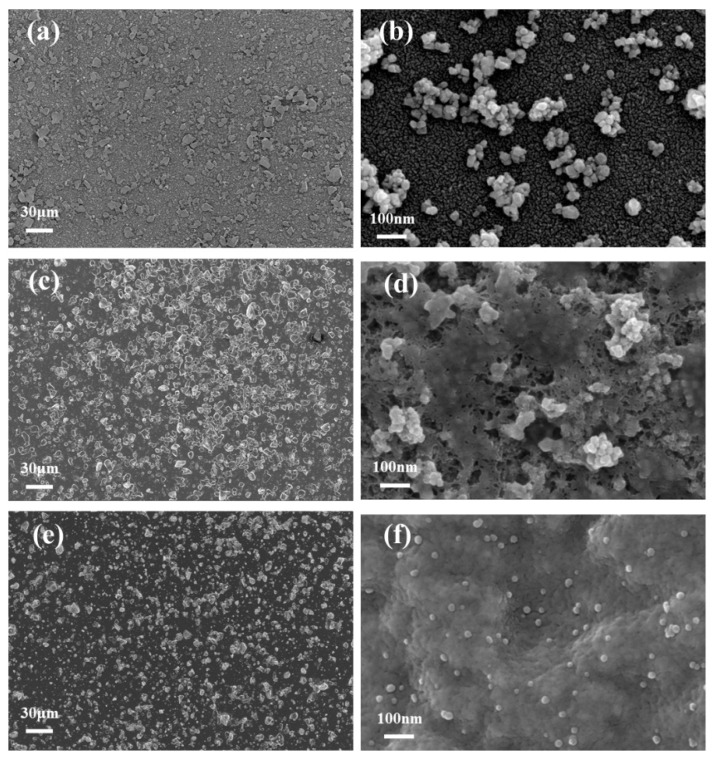
SEM images of the materials: (**a**) SEM image of CeO_2_ at low magnification; (**b**) SEM image of CeO_2_ at high magnification; (**c**) SEM image of CeO_2_/Nafion at low magnification; (**d**) SEM image of CeO_2_/Nafion at high magnification; (**e**) SEM image of AgNPs@CeO_2_/Nafion at low magnification; (**f**) SEM image of AgNPs@CeO_2_/Nafion at high magnification.

The microstructure of the materials was investigated by SEM, as shown in Figure 2. Figure 2a,b display CeO_2_ nanoparticles at different magnifications. The particles exhibit a granular morphology with non-uniform distribution and significant agglomeration on the electrode surface, which may impede electron transfer efficiency. Figure 2c illustrates the CeO_2_/Nafion composite, where Nafion disperses the CeO_2_ nanoparticles effectively, reducing agglomeration and ensuring a more uniform distribution. Figure 2d (at high magnification) demonstrates that Nafion forms a film-like structure, encapsulating CeO_2_ nanoparticles and thereby enhancing their stability. Moreover, Nafion’s strong adsorption capacity provides abundant sites for target metal ions during redox reactions. Figure 2e,f illustrate the microstructure images of AgNPs@CeO_2_/Nafion composites with varying particle sizes. A substantial amount of AgNPs distributes on the surface of CeO_2_/Nafion uniformly. The high-magnification image reveals that AgNPs exhibit a relatively uniform spherical morphology, with diameters ranging from approximately 10 to 20 nm. The incorporation of AgNPs increases the specific surface area of the electrode, providing additional catalytic active sites for the oxidation of heavy metal ions and improving electron transfer efficiency.

**Figure 3:**
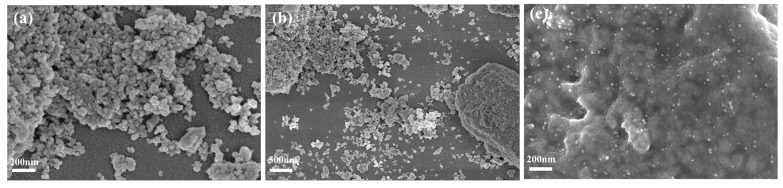
SEM images of the materials: (**a**,**b**) SEM images of AgNPs@CeO_2_ at different magnifications; (**c**) SEM image of AgNPs@CeO_2_/Nafion.

Figure 3 demonstrates the distinct differences in the microstructure of AgNPs synthesized on CeO_2_ and CeO_2_/Nafion substrates. Figure 3a,b, respectively, show the morphological features of the AgNPs@CeO_2_ composite in high-resolution images at the scales of 200 nm and 500 nm. Silver-white AgNPs are observed to aggregate, forming distinct clusters on the electrode surface, with irregular polyhedral structures. Figure 3c presents the structural morphology of the AgNPs@CeO_2_/Nafion composite at a magnification of 200 nm. After introducing Nafion perfluorosulfonic acid resin, the aggregation of AgNPs is significantly reduced, and the AgNPs exhibit highly dispersed spherical morphologies. This suggests that the CeO_2_/Nafion substrate plays a positive role in enhancing the dispersion state, controlling the particle size, and stabilizing the shape of the formed AgNPs. This result may be attributed to the sulfonic acid groups in Nafion molecules, which exert electrostatic repulsion to suppress the random aggregation of Ag^+^ ions. This leads to a more uniform concentration gradient distribution of Ag^+^ ions during deposition, thereby effectively mitigating agglomeration.

**Figure 4:**
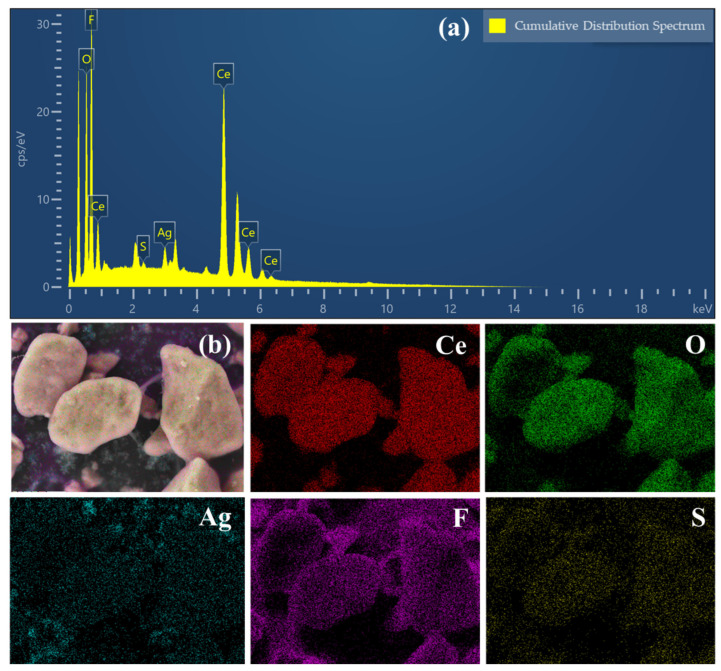
(**a**) EDX spectra of AgNPs@CeO_2_/Nafion sample; (**b**) SEM image of AgNPs@CeO_2_/Nafion sample and corresponding elemental mapping of the Ce, O, Ag, F, and S elements.

The elemental composition and distribution of the AgNPs@CeO_2_/Nafion composite were characterized by EDX, as illustrated in Figure 4. Figure 4a shows that the EDX spectrum confirms the existence of Ce, O, Ag, F, and S, with Ag accounting for 1.46 wt% and Ce for 23.66 wt%. As shown in Figure 4b, elemental mapping reveals that, in regions with a lower Nafion content, the AgNP content is also relatively reduced. Although AgNPs are observed to be more densely distributed in certain localized micro-regions, they generally exhibit a uniform spatial distribution across the sample. This morphological feature further suggests that the Nafion membrane acts as a stable substrate for the heterogeneous nucleation of AgNPs, thereby facilitating their orderly formation.

### 3.2. Electrochemical Characterization of Modified Electrodes

**Figure 5:**
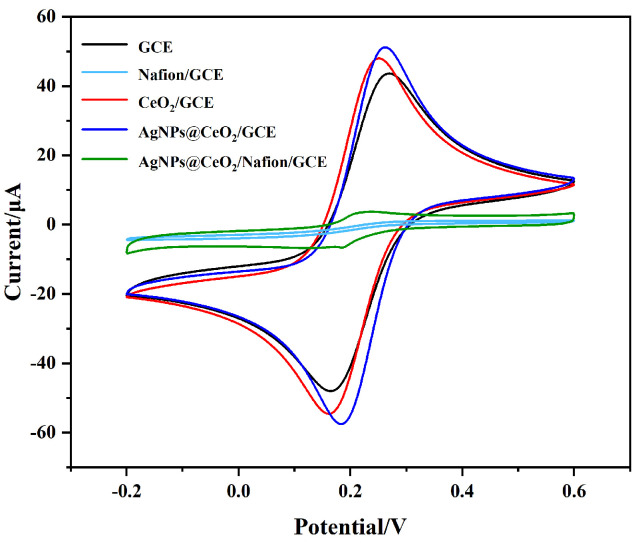
CV curves of GCE, Nafion/GCE, CeO_2_/GCE, AgNPs@CeO_2_/GCE, and AgNPs@CeO_2_/Nafion/GCE modified electrodes in 5 mM [Fe(CN)_6_]^3−^/^4−^ containing 0.1 M KCl. Conditions: 50 mV·s^−1^ scan rate, potential window from −0.2 to +0.6 V.

The electrochemical properties of the modified electrodes were assessed using CV, employing K_3_[Fe(CN)_6_] as the redox probe. Figure 5 illustrates the comparison of CV curves for five different electrodes: bare GCE, Nafion/GCE, CeO_2_/GCE, AgNPs@CeO_2_/GCE, and AgNPs@CeO_2_/Nafion/GCE. Bare GCE and CeO_2_/GCE exhibit a pair of redox peaks with oxidation peak currents of 47.92 μA and 52.97 μA, respectively. The increased current observed for CeO_2_/GCE indicates enhanced redox kinetics, which can be attributed to the oxygen vacancies and electron transfer capability of CeO_2_. The oxidation current of AgNPs@CeO_2_/GCE exhibits a further increase to 60.61 μA. This enhancement is attributed to the high conductivity and large specific surface area of AgNPs, which facilitate electron transport and catalytic activity. Nafion/GCE displays no redox peaks, likely due to the non-conductive Nafion film acting as a diffusion barrier, hindering [Fe(CN)_6_]^3−^/^4−^ from reaching the electrode surface. Additionally, the negatively charged sulfonic acid (SO_3_^−^) groups in Nafion electrostatically repel the anionic [Fe(CN)_6_]^3−^/^4−^ species, further suppressing electron transfer [33,34]. Remarkably, AgNPs@CeO_2_/Nafion/GCE not only restores but also significantly amplifies the redox currents. Despite Nafion’s poor conductivity, its strong Pb^2+^ preconcentration ability and structural stability synergize with CeO_2_’s redox activity and AgNPs’ catalytic efficiency, enabling accelerated electron transfer kinetics and amplified electrochemical signals. According to the Randles–Sevcik equation:

$$I_{P}=2.69\times 10^{5}\;A\;D^{1/2}{\;n}^{3/2}{\;v}^{1/2}\;C$$

where I_p_ is the peak current, A is the electrode area, D is the diffusion coefficient, n is the number of electron transfers, v is the scan rate, and C is the concentration of [Fe(CN)_6_]^3−^/^4−^. The electroactive surface areas of CeO_2_/Nafion/GCE and AgNPs@CeO_2_/Nafion/GCE were determined to be 0.00596 cm^2^ and 0.01161 cm^2^ on average, respectively. This suggests that the presence of AgNPs significantly enhances the effective electroactive sites of the AgNPs@CeO_2_/Nafion/GCE electrode. CV not only reflects the apparent current response at the electrode interface but also provides corroborative evidence for the successful modification of the Nafion membrane and the effective deposition of AgNPs. The primary reason for the smaller CV response current of AgNPs@CeO_2_/Nafion/GCE compared to AgNPs@CeO_2_/GCE originates from the electrostatic shielding effect of the Nafion layer on the anionic redox probe [Fe(CN)_6_]^3−^/^4−^. This observation is consistent with the characteristic charge-selective behavior of Nafion-modified electrodes reported in prior studies [33,35].

### 3.3. Detection of Pb^2+^ with Modified Electrodes

**Figure 6:**
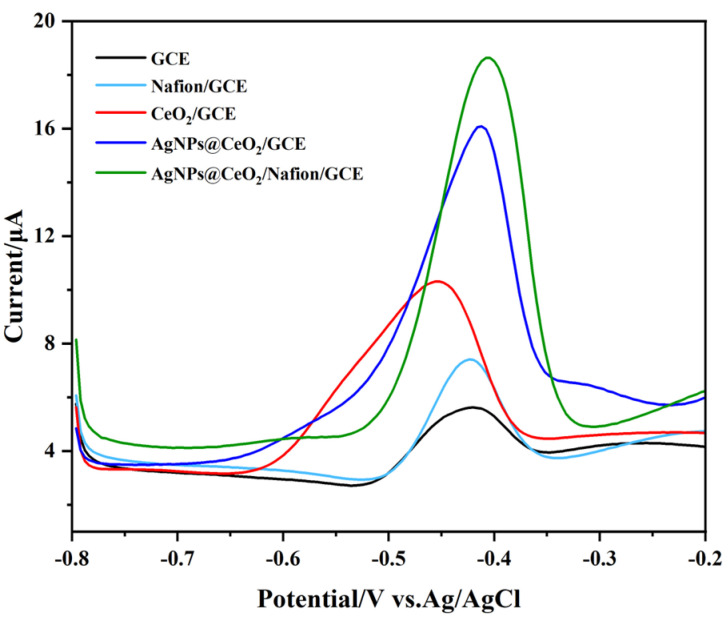
SWASV responses to 50 μg·L^−1^ Pb^2+^ on different modified electrodes: GCE, Nafion/GCE, CeO_2_/GCE, AgNPs@CeO_2_/GCE, and AgNPs@CeO_2_/Nafion/GCE in pH 4.5, 0.1 M HAc-NaAc solution. Deposition: −1.1 V for 300 s.

The response signal intensity of heavy metal ion detection by modified electrodes is influenced by multiple physicochemical factors, including interfacial electron transfer kinetic, target ion enrichment capacity, and the structural stability of functional modifiers. Figure 6 compares the SWASV responses of five different electrodes—bare GCE, Nafion/GCE, CeO_2_/GCE, AgNPs@CeO_2_/GCE, and AgNPs@CeO_2_/Nafion/GCE—when exposed to a 50 μg·L^−1^ Pb^2+^ solution. The bare GCE exhibits the lowest stripping peak current with a weak signal. When Nafion was modified on the bare electrode surface, the stripping peak current for Pb^2+^ detection was enhanced by a factor of two. The CV diagram above demonstrates that, despite Nafion’s relatively low conductivity, it still promotes the response signal of Pb^2+^. This is attributed to the fact that the sulfonic acid groups in the side chains of Nafion can enhance the enrichment of Pb^2+^ through electrostatic attraction, thereby providing more heavy metal ions for the redox reaction on the electrode surface. The peak current of CeO_2_/GCE is also significantly higher than that of the bare electrode. This improvement can be attributed to the fact that CeO_2_ nanoparticles provide a high specific surface area for Pb^2+^ preconcentration and facilitate electron transfer through their oxygen vacancy-mediated redox activity. When AgNPs were introduced into CeO_2_/GCE, it was observed that the stripping peak current of Pb^2+^ on AgNPs@CeO_2_/GCE increased significantly, and the peak shape became more defined. This suggests that the AgNPs, with their high electrical conductivity, not only improved the charge transfer efficiency at the electrode surface but also effectively increased the specific surface area of the electrode. AgNPs@CeO_2_/Nafion/GCE exhibited the highest peak current and the narrowest half-peak width among all tested electrodes. In conjunction with the aforementioned material characterization results, the incorporation of Nafion facilitated a more uniform distribution of CeO_2_ and AgNPs on the electrode surface. The synergistic interaction among the three materials resulted in the optimal response signal for Pb^2+^ detection. In addition, Nafion, as a polymer membrane, exhibits excellent support properties and effectively mitigates the shedding of AgNPs@CeO_2_ during the detection process, thereby significantly enhancing the stability of the modified material. Therefore, the AgNPs@CeO_2_/Nafion/GCE was selected as the modified electrode for the detection of Pb^2+^.

### 3.4. Optimization of Experimental Conditions

To achieve optimal performance for Pb*^2^*^+^ detection, the experimental parameters were systematically optimized using a one-variable-at-a-time approach.

#### 3.4.1. Preparation Conditions of the Modified Electrode

**Figure 7:**
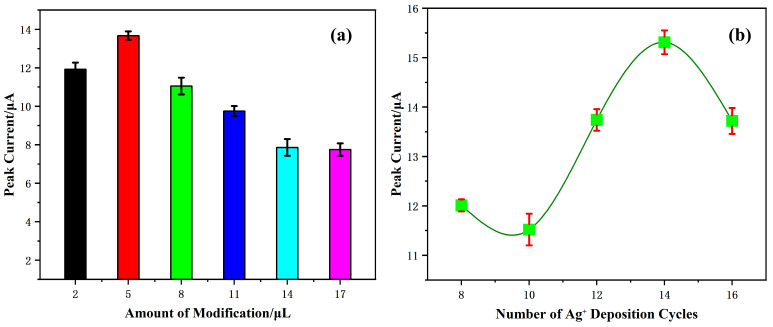
Effects of (**a**) modification amount of CeO_2_/Nafion material; (**b**) the number of deposition cycles of silver on SWASV stripping currents toward 50 μg·L^−1^ Pb^2+^ in pH 4.5, 0.1 M HAc-NaAc solution. Error bars represent standard deviation (SD) from triplicate experiments.

First, the electrode preparation conditions were investigated. The modification amount of electrode materials significantly influences the analytical performance of the sensor. The relationship between varying drop-coating amounts of CeO_2_/Nafion and the stripping peak current of Pb^2+^ was investigated using the SWASV method. As illustrated in Figure 7a, The peak current reaches its maximum when the modification amount is 5 μL. Beyond this volume, the peak current exhibits a gradual decline. Thus, 5 μL was selected as the optimal modification volume.

The number of Ag^+^ deposition cycles directly affects the quantity of AgNPs formed, which in turn affects the sensitivity of the test. As shown in Figure 7b, the stripping peak currents for Pb^2+^ detection vary with the number of deposition cycles. The current response reaches its maximum at 14 deposition cycles. Consequently, 14 cycles were chosen for subsequent experiments.

#### 3.4.2. Testing Conditions of the Modified Electrode

Subsequently, the testing conditions were optimized, including electrolyte type, pH, deposition time, and potential.

**Figure 8:**
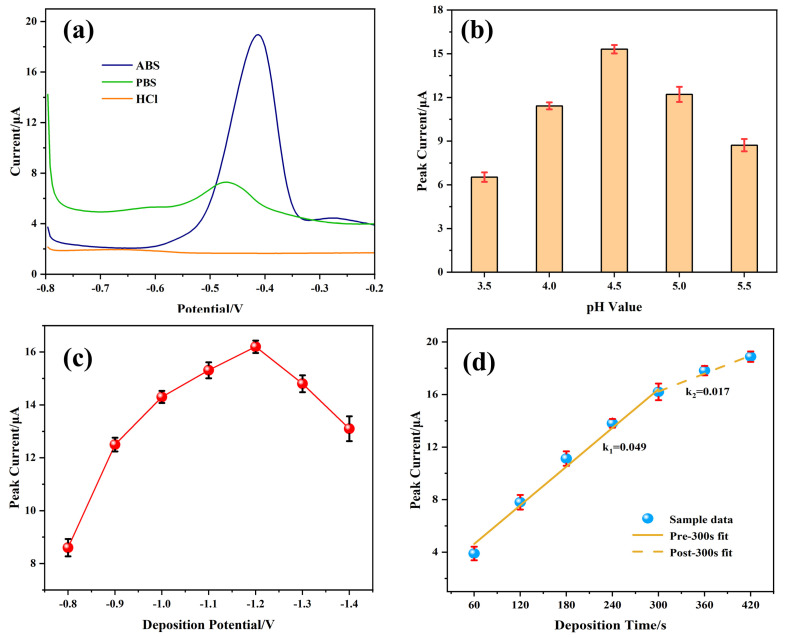
Effects of (**a**) type of supporting electrolytes; (**b**) pH value; (**c**) deposition potential; (**d**) deposition time on the SWASV stripping currents of 50 μg·L^−1^ Pb^2+^. Error bar: n = 3.

The voltametric responses of the fabricated sensor were systematically investigated in three electrolytes: 0.1 M ABS at pH 4.5, 0.1 M PB at pH 4.5, and 0.1 M HCl adjusted to pH 4.5 using NaOH. This study aimed to identify the most suitable stripping solvent for Pb^2+^ detection. As shown in Figure 8a, ABS produces the highest Pb*^2^*^+^ oxidation peak current, while HCl and PB yield minimal signals. These results confirm ABS as the optimal supporting electrolyte.

The electrolyte pH critically influences the stripping current. Figure 8b shows that the Pb*^2^*^+^ peak current increases as pH rises from 3.5 to 4.5 but declines at higher pH values. At low pH values, hydronium ions (H_3_O^+^) and Pb^2+^ compete for reaction sites, leading to hydrogen evolution on the electrode surface, which impairs the preconcentration of Pb^2+^ [36]. Conversely, at high pH levels, Pb*^2^*^+^ hydrolysis reduces dissolution peak signal. Therefore, after comprehensive consideration, the optimal pH value of the supporting electrolyte is determined to be 4.5.

The deposition potential also impacts detection peak. Figure 8c indicates that a deposition potential of −1.2 V maximizes the Pb*^2^*^+^-stripping current. Potentials more negative than −1.2 V increase hydrogen evolution, which hinders Pb^2+^ accumulation [37]. Consequently, −1.2 V was determined to be the optimal deposition voltage.

Finally, the optimization of deposition time (Figure 8d) reveals that increasing the deposition duration from 60 s to 300 s enhances the Pb*^2^*^+^ signal due to increased surface loading. Beyond 300 s, the current reaches a plateau, indicating the saturation of active sites. Additionally, a prolonged deposition time can lead to a certain extent of damage to the modified materials [38]. Therefore, 300 s was chosen as the optimal deposition time.

### 3.5. Analytical Performance

**Figure 9:**
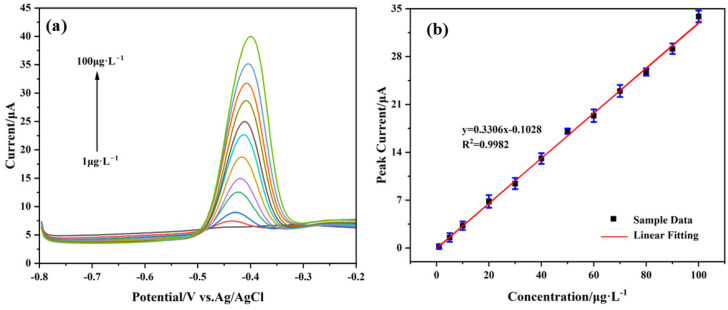
(**a**) SWASV responses of the AgNPs@CeO_2_/Nafion/GCE for the analysis of Pb^2+^ over a concentration range of 1–100 μg·L^−1^ in pH 4.5, 0.1 M HAc-NaAc solution. (**b**) Corresponding linear calibration of peak current. Deposition potential, −1.2 V; deposition time, 300 s. Error bar: n = 3.

Under optimal conditions, the analytical performance of the AgNPs@CeO_2_/Nafion/GCE for Pb^2+^ detection was evaluated using the SWASV method. As shown in Figure 9a,b, within the concentration range of 1 to 100 μg·L^−1^, the stripping peak current of Pb^2+^ exhibits a linear relationship with its concentration. The linear regression analysis yielded the equation: y = 0.3306x − 0.1028 (R^2^ = 0.9982), indicating a sensitivity of 0.3273 µA·µg^−1^·L and a detection limit (LOD) of 0.17 μg·L^−1^ (S/N = 3). Notably, this LOD is significantly lower than the World Health Organization (WHO) permissible threshold of 10 μg·L^−1^ for Pb^2+^ in drinking water. A comparative analysis with previously reported Pb^2+^ sensors (Table 1) shows that the AgNPs@CeO_2_/Nafion sensor developed in this study demonstrates superior performance compared to most similar sensing systems in terms of the detection limit for Pb^2+^. Although its detection limit for lead ions is slightly higher than that of Bi/Nafion/RGO-GNPs/GCE, it exhibits significant comprehensive advantages in practical application scenarios. The linear sensitivity of this sensor reaches 0.3273 μA·μg^−1^·L, which is approximately 20% higher than that of Bi-based sensors, while the cost of a single electrode is substantially reduced. Furthermore, by utilizing biocompatible CeO_2_ material, the risk of Bi^3+^ ion dissolution associated with Bi-based sensors is effectively mitigated. Through the synergistic optimization of sensitivity, cost efficiency, and environmental safety, this study offers a more engineering-oriented and practical solution for on-site monitoring of heavy metals in water.

### 3.6. Anti-Interference, Repeatability, and Reproducibility Studies

#### 3.6.1. Anti-Interference Capability

Anti-interference performance is a critical indicator for evaluating modified electrodes used in the detection of real water samples. Metal ions and organic compounds may interfere with Pb^2+^ detection, thereby compromising the accuracy of the results. To evaluate the interference resistance of the AgNPs@CeO_2_/Nafion/GCE in Pb^2+^ detection, this study systematically selected several metal ions, small-molecule organic compounds, and common surfactants to conduct interference experiments.

**Figure 10:**
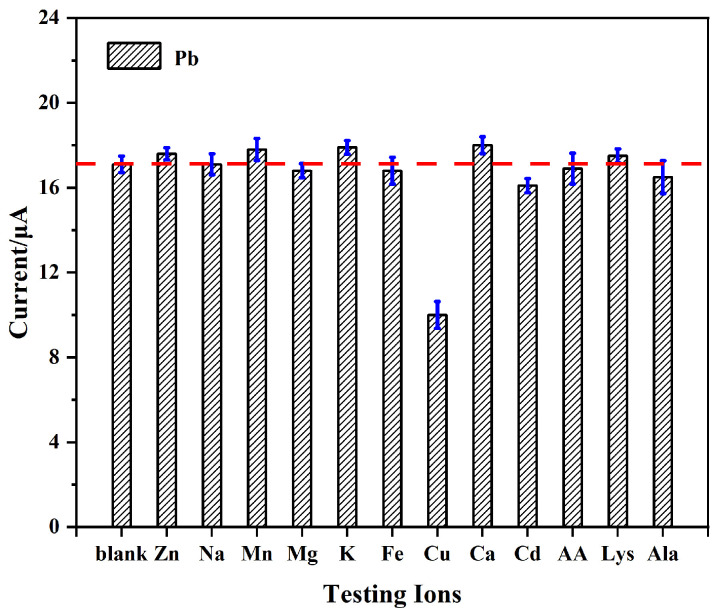
Effect of ionic and organic interference on the detection of 50 μg·L^−1^ Pb^2+^. Error bar: n = 3.

The electrochemical analysis of Pb^2+^ was conducted by sequentially adding 20 times the concentration of potential interfering ions (Zn^2+^, Na^+^, Mn^2+^, Mg^2+^, K^+^, Fe^3+^, Cu^2+^, Ca^2+^, and Cd^2+^) and small organic molecules (AA, Lys, Ala) to a solution containing 50 μg·L^−1^ of Pb^2+^. As shown in Figure 10, the addition of these interferents causes negligible variations (<5% relative standard deviation, RSD) in the Pb^2+^-stripping current, with the exception of Cu^2+^. The interference from Cu^2+^ is likely attributed to competitive adsorption with Pb^2+^ at the active sites during electrodeposition, thereby reducing the preconcentration efficiency of Pb^2+^. Previous studies have suggested that introducing ferrocyanide ions to form stable Cu^2+^–ferrocyanide complexes can mitigate this interference [43].

**Figure 11:**
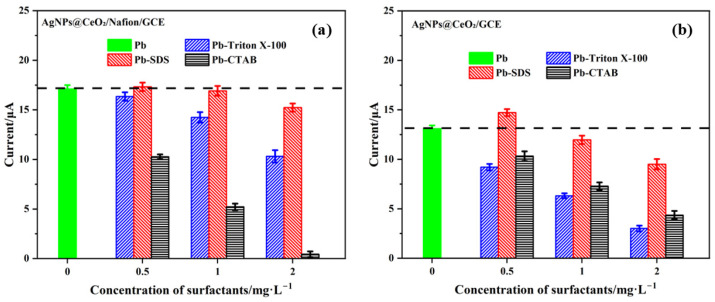
Effect of surfactant concentration on Pb^2+^ (50 μg·L^−1^) detection performance: (**a**) AgNPs@CeO_2_/Nafion/GCE and (**b**) AgNPs@CeO_2_/GCE electrodes in 0.1 M HAc-NaAc solution (pH 4.5) with Triton X-100, SDS, and CTAB. Error bar: n = 3.

When surfactants are present in water samples, their molecules tend to adsorb onto the electrode surface, thereby interfering with the accurate detection of Pb^2+^. This study evaluated the anti-interference performance of AgNPs@CeO_2_/Nafion/GCE modified electrodes for Pb^2+^ detection in the presence of three typical surfactants—Triton X-100 (non-ionic), SDS (anionic), and CTAB (cationic)—at various concentrations. The detailed test results are presented in Figure 11.

As shown in Figure 11a, the AgNPs@CeO_2_/Nafion/GCE sensor demonstrated satisfactory anti-interference capability against low-concentration Triton X-100 (≤1.0 mg·L^−1^), with the current response retention rate of Pb^2+^ exceeding 90%. However, when the Triton X-100 concentration increased to 2.0 mg·L^−1^, a notable decline in Pb^2+^ current response was observed. In SDS interference experiments, the sensor exhibited excellent stability, showing minimal fluctuation in Pb^2+^ oxidation peak current within the 0.5–2.0 mg·L^−1^ concentration range. In contrast, distinct interference effects were observed with CTAB. Even at a low CTAB concentration of 0.5 mg·L^−1^, the Pb^2+^-stripping peak signal was significantly suppressed, indicating the sensor’s insufficient anti-interference capability toward cationic surfactants. The anti-interference performance of AgNPs@CeO_2_/GCE toward three surfactants was systematically evaluated. As illustrated in Figure 11b, the unmodified AgNPs@CeO_2_/GCE sensor demonstrated limitations in its interference resistance performance. Compared with the Nafion-modified counterpart, its tolerance thresholds for Triton X-100 and SDS were found to be lower. Although showing relative advantage in CTAB interference tests, the AgNPs@CeO_2_/GCE still failed to maintain effective anti-interference capability when CTAB concentration exceeded 1.0 mg·L^−1^. The experimental results demonstrate that the Nafion modification layer significantly enhances the sensor’s anti-interference capability against anionic and nonionic surfactants through its unique cation-selective permeability and electrostatic repulsion mechanisms. However, its shielding effect on cationic surfactants remains limited. The experimental results confirm the alignment with the synergistic effects of cation-sieving behavior and electrostatic repulsion derived from sulfonic acid groups in the Nafion-modified layer [29,44]. Additionally, these findings are consistent with the interference patterns of surfactants on heavy metal ion detection as reported in prior studies [43,45,46]. When cationic surfactants are present in the system, pre-treatment methods can be employed to eliminate these surfactants prior to Pb^2+^ detection.

#### 3.6.2. Repeatability and Reproducibility

**Figure 12:**
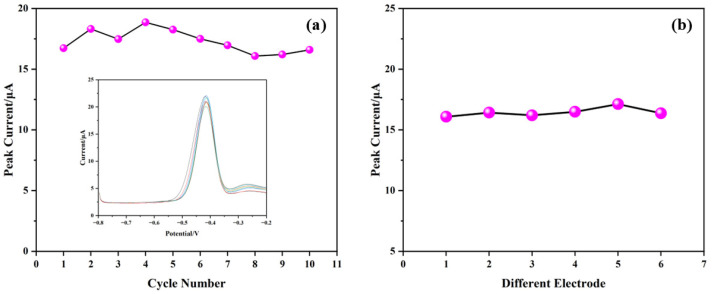
Evaluation of (**a**) the repeatability of AgNPs@CeO_2_/Nafion/GCE with 10 consecutive measurements; (**b**) the reproducibility of AgNPs@CeO_2_/Nafion/GCE with six fabricated sensors.

The repeatability of the sensor was tested by performing ten consecutive SWASV measurements on the same electrode in a 0.1 M HAc-NaAc buffer solution (pH 4.5) containing 50 μg·L^−1^ Pb^2+^. Figure 12a shows the stripping peak currents for these measurements, with an RSD of 5.46%, confirming good repeatability. The inset in Figure 12a displays the voltammetric curves corresponding to these trials. To ensure reproducibility, six independently fabricated electrodes were tested under identical conditions. As shown in Figure 12b, the RSD of the Pb^2+^-stripping currents among the six electrodes is 2.20%, demonstrating good batch-to-batch consistency.

Table 1 compares the repeatability and reproducibility of the AgNPs@CeO_2_/Nafion/GCE sensor with other Pb^2+^ detection sensors. Although the newly developed Pb^2+^ sensor exhibits a disparity in repeatability compared to those reported in the literature, its reproducibility demonstrates superior performance over existing counterparts. This characteristic highlights the sensor’s enhanced reliability across different operational batches.

### 3.7. Analysis of Real Water Samples

The AgNPs@CeO_2_/Nafion/GCE sensor was used to detect Pb^2+^ in real water samples. Prior to sensor analysis, water samples were pre-treated following the procedure described in Section 2.5 and subsequently subjected to SWASV analysis under the optimized conditions. As shown in Table 2, the concentrations of Pb^2+^ detected in the surface layer of the Xiangjiang River and in rainwater were 3.73 ± 0.36 μg·L^−1^ and 1.85 ± 0.76 μg·L^−1^, respectively. No detectable response signal for Pb^2+^ was observed in either tap water or mineral water, indicating either the absence of Pb^2+^ or its concentration being below the detection limit of the electrode system. To validate the accuracy, spike recovery tests were conducted by adding 10 and 20 μg·L^−1^ of Pb^2+^ to the samples. The recovery rates for the water samples ranged from 93.7% to 110.3% (n = 6), indicating the acceptable accuracy of the analytical method within the specified limits. These results were further validated by ICP-MS, thereby confirming excellent consistency between the two measurement methods. These results demonstrate that the AgNPs@CeO_2_/Nafion/GCE sensor is a reliable method for detecting Pb^2+^ in real water samples.

## 4. Conclusions

In this study, an innovative nano-CeO_2_/Nafion composite system was utilized to modify GCE. Nano-CeO_2_ and Nafion served as carriers, and a highly dispersed AgNP modification layer was electrochemically deposited onto the electrode surface. Consequently, an environmentally friendly lead ion electrochemical sensor was successfully developed. The composites were characterized using XRD, SEM, and various electrochemical techniques, which confirmed their detection capability for Pb^2+^. After optimization and testing by SWASV, the detection limit of the sensor for Pb^2+^ was as low as 0.17 μg·L^−1^. The recovery rate in the detection of real water samples ranged from 93.7% to 110.3%. Notably, this sensor exhibited excellent anti-interference capability against most coexisting heavy metal ions (except copper ions), small-molecule organic interferents, and specific concentrations of nonionic/anionic surfactants. Furthermore, the sensor demonstrated good repeatability and batch-to-batch consistency, which provides a critical technical guarantee for practical application and promotion. Additionally, in this study, the low-cost combination of CeO_2_ and Nafion not only reduced the cost of modification materials but also ensured detection performance, highlighting its potential for large-scale application.

## Figures and Tables

**Table 1:** Comparison of Pb^2+^ performance detected by different types of electrochemical sensors.

Electrodes	Technique	Linear Range (μg·L^−1^)	Detection Limit (μg·L^−1^)	Repeatability (RSD%)	Reproducibility (RSD%)	Reference
Graphene/Nafion/GCE	DPASV	4.14–5000	1.06	2.0 (n ^a^ = 5)	N/A ^b^	[39]
Bi/Nafion/RGO-GNPs/GCE	SWASV	1–90	0.12	1.25 (n = 6)	N/A	[30]
Fe-OSA/Nafion GCE	DPASV	8.3–2776	1.5	N/A	N/A	[38]
ZnCo ZLDH@CeO_2_/NF	DPASV	21–6200	1.9	N/A	N/A	[40]
AgNF@GCE	SWASV	10–700	0.74	2.8 (n = 8)	N/A	[41]
Nafion/CLS/PGR/GCE	DPASV	10–1000	2.1	3.63 (n = 8)	3.85 (n = 8)	[29]
PtNPs/Au	SWV	21~104	10	0.6	N/A	[42]
AgNPs@CeO_2_/Nafion/GCE	SWASV	1–100	0.17	5.46 (n = 10)	2.2 (n = 6)	This work

^a^: Number of measurements. ^b^: Not available.

**Table 2:** Comparison results of Pb^2+^ in real water samples (n = 6).

Samples	Added (μg·L^−1^)	Found by Proposed Method ^a^ (μg·L^−1^)	Found by ICP-MS ^a^ (μg·L^−1^)	Recovery ^b^ (%)
Tap water	0	N.D.	N.D.	—
	10	11.03 ± 0.57	9.89 ± 0.42	110.3
	20	21.26 ± 0.69	20.79 ± 0.31	106.3
Xiangjiang River	0	3.73 ± 0.36	3.54 ± 0.16	—
	10	13.89 ± 0.51	14.07 ± 0.29	101.6
	20	22.57 ± 0.42	23.98 ± 0.24	94.2
Rainwater	0	1.85 ± 0.76	2.01 ± 0.39	—
	10	12.64 ± 0.42	12.33 ± 0.23	107.9
	20	21.03 ± 0.53	22.17 ± 0.27	95.9
Mineral water	0	N.D.	N.D.	—
	10	10.06 ± 0.43	9.91 ± 0.38	100.6
	20	18.73 ± 0.64	21.02 ± 0.26	93.7

N.D.: Not detected. ^a^: Mean ± 95% confidence interval (CI), n = 6. ^b^: Recovery of measurements are calculated from the ratio of [Found]/[Added].

## Data Availability

Data are contained within the article.
